# The Streptococcus agalactiae Stringent Response Enhances Virulence and Persistence in Human Blood

**DOI:** 10.1128/IAI.00612-17

**Published:** 2017-12-19

**Authors:** Thomas A. Hooven, Andrew J. Catomeris, Maryam Bonakdar, Luke J. Tallon, Ivette Santana-Cruz, Sandra Ott, Sean C. Daugherty, Hervé Tettelin, Adam J. Ratner

**Affiliations:** a Department of Pediatrics, Columbia University, New York, New York, USA; b Department of Pediatrics, New York University, New York, New York, USA; c Institute for Genome Sciences, University of Maryland, Baltimore, Maryland, USA; d Department of Microbiology, New York University, New York, New York, USA; University of Illinois at Chicago

**Keywords:** Streptococcus agalactiae, bloodstream infections, Tn-seq

## Abstract

Streptococcus agalactiae (group B Streptococcus [GBS]) causes serious infections in neonates. We previously reported a transposon sequencing (Tn-seq) system for performing genomewide assessment of gene fitness in GBS. In order to identify molecular mechanisms required for GBS to transition from a mucosal commensal lifestyle to bloodstream invasion, we performed Tn-seq on GBS strain A909 with human whole blood. Our analysis identified 16 genes conditionally essential for GBS survival in blood, of which 75% were members of the capsular polysaccharide (*cps*) operon. Among the non-*cps* genes identified as conditionally essential was *relA*, which encodes an enzyme whose activity is central to the bacterial stringent response—a conserved adaptation to environmental stress. We used blood coincubation studies of targeted knockout strains to confirm the expected growth defects of GBS deficient in capsule or stringent response activation. Unexpectedly, we found that the *relA* knockout strains demonstrated decreased expression of β-hemolysin/cytolysin, an important cytotoxin implicated in facilitating GBS invasion. Furthermore, chemical activation of the stringent response with serine hydroxamate increased β-hemolysin/cytolysin expression. To establish a mechanism by which the stringent response leads to increased cytotoxicity, we performed transcriptome sequencing (RNA-seq) on two GBS strains grown under stringent response or control conditions. This revealed a conserved decrease in the expression of genes in the arginine deiminase pathway during stringent response activation. Through coincubation with supplemental arginine and the arginine antagonist canavanine, we show that arginine availability is a determinant of GBS cytotoxicity and that the pathway between stringent response activation and increased virulence is arginine dependent.

## INTRODUCTION

Streptococcus agalactiae (group B Streptococcus [GBS]) is a common adult intestinal and vaginal commensal that also causes neonatal sepsis, pneumonia, and meningitis (1, 2). It is the leading cause of infectious neonatal mortality in the United States (3). Enhanced understanding of the host-pathogen interactions that permit GBS to convert from a commensal to an invasive lifestyle would help advance the development of improved preventative and therapeutic approaches.

GBS expresses numerous virulence factors, although there is variability in expression among individual strains (4–9). One well-studied virulence factor is the pigmented ornithine-rhamnopolyene β-hemolysin/cytolysin (βHC; also referred to as granadaene) (10). Although its exact molecular mechanism is not understood, βHC has been shown to be cytotoxic to a variety of human cells and to contribute to virulence in several animal models of disease (11–15). There is considerable variability in βHC expression among GBS strains, even among pathogenic strains (16). Within individual strains, changes in the environment—such as shifts in pH or temperature—affect βHC expression (17, 18).

We recently reported the development and validation of a transposon sequencing (Tn-seq) method for performing unbiased, whole-genome identification of essential or conditionally essential (CE) GBS genes (19). Tn-seq uses next-generation sequencing (NGS) of a saturated transposon mutant library to compare transposon insertions in library bacteria grown under experimental conditions to control library outgrowth. Genes with decreased transposon insertion densities after the experimental exposure are likely essential for bacterial growth under that condition; the decrease in transposon insertions detected indicates that mutants bearing knockouts of those genes, which were present in the starting library, have died off (20, 21).

In this study, we apply our Tn-seq method to identifying gene products necessary for GBS survival in human whole blood. We show that the GBS polysaccharide capsule is CE for survival in blood, as is RelA, a ribosome-associated GTP pyrophosphokinase. RelA is a central effector of the bacterial stringent response (SR), a conserved, global transcriptional adaptation to environmental stress (22–24). Using transcriptomics and confirmatory coincubation studies, we show that in addition to promoting GBS persistence in human blood, activation of the stringent response enhances βHC expression through an arginine-mediated pathway and transcription of genes involved in arginine metabolism is implicated in βHC expression variability among different GBS strains.

## RESULTS

### GBS Tn-seq in whole blood identifies capsule and RelA as conditionally essential.

In order to maximize the resolution of our Tn-seq method, we combined three Tn-seq-compatible GBS transposon mutant libraries to generate a pooled master library in a background of the pathogenic GBS serotype Ia strain A909. The generation of the libraries and our basic Tn-seq methodology have been described previously (19).

We performed library outgrowth for 6 h in five samples of fresh whole blood from three healthy adult volunteers and one control condition of selective medium. Then, bacterial genomic DNA was purified from each sample, digested with MmeI, ligated to barcoded adapters, and used as the template for PCR. The resultant amplicons were purified and sequenced by NGS. The reads were trimmed of all transposon and adapter sequences, leaving only 16-nucleotide (nt) GBS genomic-DNA sequences, which were aligned to the A909 genome. These alignments were then analyzed using ESSENTIALS, an open-access Tn-seq bioinformatics tool that compares experimental and control alignments, in order to identify CE genes from the experimental condition (25).

**FIG 1:**
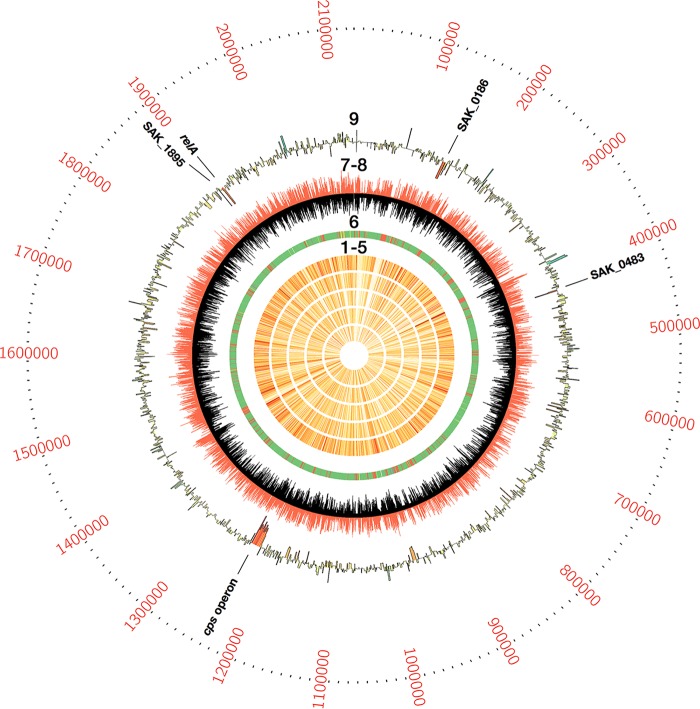
Circos plot of GBS whole blood Tn-seq results. Rings 1 to 5 show detected transposon insertion counts per gene (white, lower; red, higher) for the five blood coincubation samples analyzed. Ring 6 shows baseline fitness previously determined for each gene (green, nonessential; red, essential; yellow, critical; gray, undetermined) (19). Rings 7 and 8 show log-transformed unique transposon insertion counts at each TA dinucleotide site for the control coincubation sample (ring 7, black) and the mean of all five experimental samples (ring 8, red). Ring 9 illustrates LFC for each gene, heat mapped for the adjusted *P* value assigned by ESSENTIALS (red, lower; green, higher). The five loci that had LFC values below the CE threshold are labeled.

Genomewide results from our Tn-seq analysis are presented as a Circos plot in Fig. 1 (26). After passage through blood or, in the case of the control sample, tryptic soy broth (TSB) with erythromycin (Erm) (TSB Erm) selection, sequencing of DNA from our six samples identified 62,217 unique flanking transposon insertion sites.

The number of transposon insertions detected within each gene was highly reproducible among our five blood coincubation replicates (Fig. 1, rings 1 to 5). Comparison between TA site insertions detected in the control, under the broth-only coincubation condition, and in the five blood coincubation samples (Fig. 1, rings 7 to 8) identified several regions of statistically significant divergence, where fewer TA site insertions were detected in the blood coincubation samples than in the control sample, indicating that these regions were CE for GBS survival in human whole blood (Fig. 1, ring 9).

**FIG 2:**
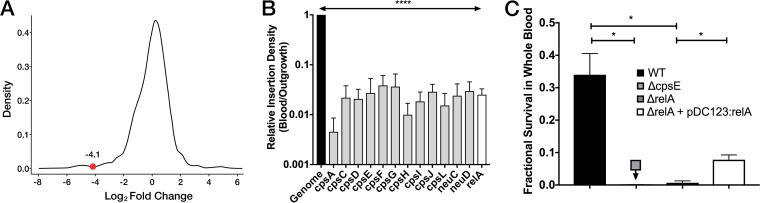
Conditionally essential genes for GBS whole blood survival. (A) Density versus LFC plot generated by ESSENTIALS, with the LFC threshold between nonessential and CE genes (−4.1) indicated. (B) Mean unique transposon insertions within CE genes of the *cps* operon (gray bars) and *relA* (white bar) detected after blood coincubation relative to those in control outgrowth culture, normalized to the genomewide ratio of experimental to control transposon insertion detections (black bar). ****, *P* < 0.001, exact test based on the negative binomial distribution model in EdgeR incorporated in the ESSENTIALS package. (C) Fractional survival of A909 WT, Δ*cpsE*, Δ*relA*, and Δ*relA*+pDC123:*relA* after coincubation with heparinized whole blood. Each coincubation was repeated at least three times. *, *P* < 0.05, *t* test with Bonferroni correction for multiple comparisons. Data represent mean values of all replicates, with error bars indicating standard errors of the means.

ESSENTIALS generates a plot of kernel function density versus log_2_-fold change (LFC; actual versus expected transposon insertions) for the genome (25). This plot provides a visualization of the relative number of genes that have fewer than expected transposon insertions in the experimental data set. The LFC value at the local minimum between the rightmost peak (which represents genes that have approximately the expected number of insertions) and the leftmost peak (representing genes with fewer than expected transposon insertions) can be used as a cutoff between genes that are putatively dispensable and those that are CE under the experimental growth condition. In our experiment, the leftmost peak—representing CE genes for blood coincubation—was short, indicating that among genes that are nonessential for growth in medium, only a small set are CE for GBS survival in blood (Fig. 2A). Figure 1, ring 9, shows the ESSENTIALS LFC values for all genes in the A909 genome. Genomewide Tn-seq data are presented in Data Set S1 in the supplemental material.

Table 1 lists the 16 GBS genes with LFC values below the cutoff of −4.1 identified by ESSENTIALS as the threshold between dispensable and CE genes. Of these, 75% are part of the capsular polysaccharide synthesis (*cps*) operon (27). The relative paucity of transposon insertions in the *cps* operon is visible in Fig. 1, ring 9. The gene with the lowest LFC score was *cpsA*, which encodes an important regulator of capsule synthesis and whose proper function is known to be important in promoting GBS survival in human blood (28). Of the four remaining genes with subthreshold LFC values, SAK_0483 encodes an R3H domain-containing protein that is predicted to interact with single-stranded DNA or RNA but has not been studied experimentally (29). SAK_1895 encodes a predicted carbohydrate transporter subunit. SAK_0186, which encodes the IgA-binding β antigen, is found in serotype Ia, Ib, II, and some serotype III GBS strains (30, 31). Its upregulation in response to exposure to human blood, serum, and conditions associated with fetal infection has been described, as has its putative role in virulence (32–35).

**TABLE 1:** GBS gene products conditionally essential for survival in human blood and corresponding ESSENTIALS Log_2_ FC values

Gene locus	Gene product^*a*^	Log_2_ FC
SAK_1262	**Regulatory protein CpsA**	−7.34
SAK_1255	**Capsular polysaccharide synthesis protein CpsH**	−6.24
SAK_1251	**Polysaccharide biosynthesis protein CpsL**	−5.65
SAK_0483	R3H domain-containing protein	−5.64
SAK_1254	**Capsular polysaccharide biosynthesis protein**	−5.42
SAK_1259	**Tyrosine-protein kinase CpsD**	−5.23
SAK_1260	**Capsular polysaccharide biosynthesis protein CpsC**	−5.15
SAK_1249	**UDP-N-acetylglucosamine-2-epimerase NeuC**	−5.03
SAK_1900	GTP pyrophosphokinase RelA	−4.97
SAK_1895	PTS system transporter subunit IIA	−4.92
SAK_1258	**Glycosyl transferase CpsE**	−4.83
SAK_1253	**Capsular polysaccharide biosynthesis protein CpsJ**	−4.76
SAK_1248	**NeuD protein**	−4.70
SAK_0186	IgA-binding β antigen	−4.38
SAK_1256	**Polysaccharide biosynthesis protein CpsG**	−4.36
SAK_1257	**Polysaccharide biosynthesis protein CpsF**	−4.32

a The products of genes in the capsular polysaccharide locus are in boldface.

### Coincubation studies validate the Tn-seq prediction that capsule and RelA are conditionally essential for GBS survival in blood.

SAK_1900 encodes the GTP pyrophosphokinase RelA, which is a central mediator of the bacterial SR, a conserved transcriptional adaptation to environmental stress (24). RelA is a ribosome-associated enzyme that detects stalled protein translation, which can be the result of depleted micronutrients, antibiotic exposure, or immunologic pressure, such as exposure to antimicrobial peptides (23, 36, 37). In response to stalled translation, RelA phosphorylates GTP to generate the alarmone molecules guanosine tetra- and pentaphosphate [(p)ppGpp], which have been shown to act on multiple intracellular targets and second messengers, triggering global transcriptional changes, metabolic adjustment, and in some bacterial species, increased virulence (38–44).

Since studies of the GBS SR have not been reported and since it is a known mediator of virulence in other pathogenic bacteria, we decided to pursue further investigation of this pathway.

To validate the predictions made by our Tn-seq experiments, we focused on the roles of the *cps* operon and RelA. The relative transposon insertion densities in CE *cps* operon genes and *relA* are shown in Fig. 2B; all were significantly below the genomewide insertion density. We generated Δ*cpsE* and Δ*relA* knockout (KO) strains in an A909 background and complemented the Δ*relA* strain with the full-length *relA* coding sequence in *trans* (Δ*relA*+pDC123:*relA*). In whole blood coincubation studies with the Δ*cpsE* and Δ*relA* strains, we observed the expected survival impairment, which was partially rescued by complementation in the case of the Δ*relA* strain (Fig. 2C).

### SR activation increases GBS βHC expression.

**FIG 3:**
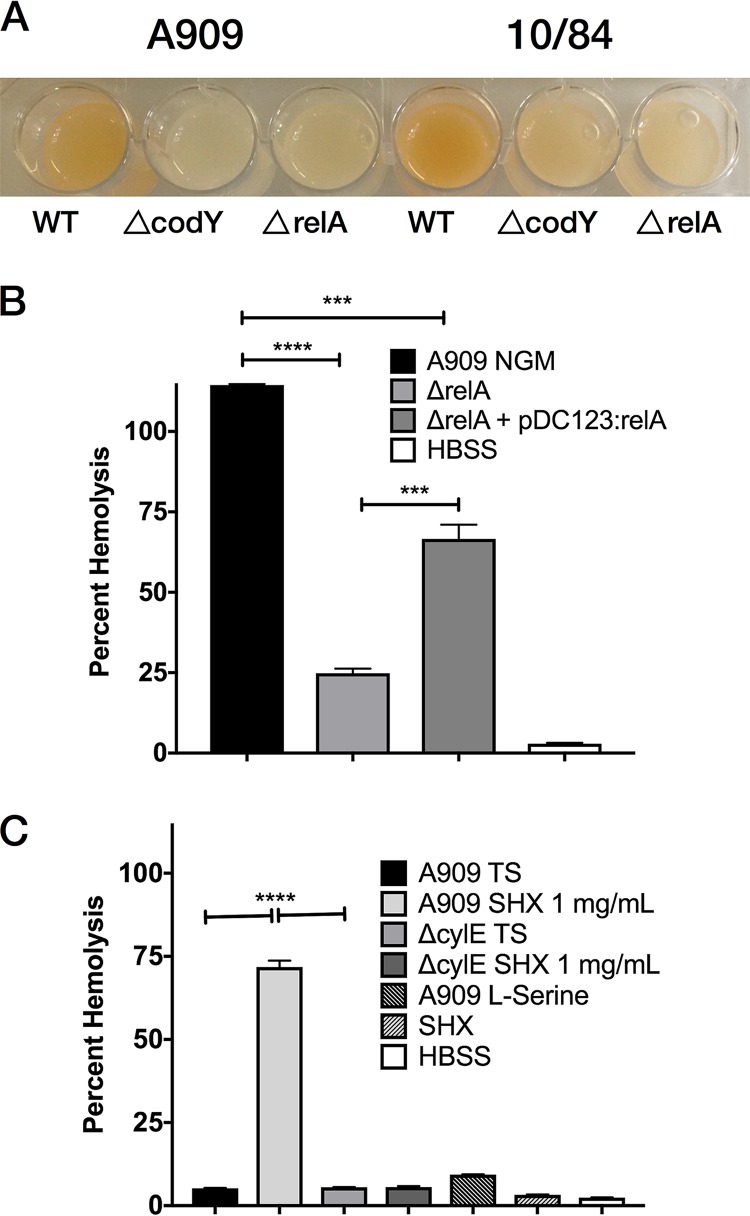
The stringent response modulates GBS βHC expression and hemolytic activity. (A) A909 and 10/84 WT and SR mutants were grown overnight in new Granada medium with appropriate antibiotic selection. The cultures were normalized for OD_600_ and volume, pelleted, resuspended in 100 μl PBS, and photographed in a 96-well plate. (B) A909 Δ*relA* shows decreased hemolysis relative to the results for the WT and Δ*relA*+pDC123:*relA* strains. (C) WT A909 grown under SR-activating conditions with supplemental SHX shows enhanced hemolysis over bacteria grown in TSB or A909 Δ*cylE*, which does not produce βHC. All hemolysis experiments were performed in triplicate and repeated at least twice. The percentage of hemolysis is relative to the result for a 1% Triton X-100 positive-control solution. Histograms show mean values, with error bars illustrating standard errors of the means. ***, *P* < 0.005, and ****, *P* < 0.0001, *t* test with Bonferroni correction for multiple comparisons.

We observed that Δ*relA* colonies were less pigmented than wild-type (WT) colonies, suggesting decreased βHC expression. The effect was especially pronounced when the KO strains were grown in pigment-enhancing new Granada medium (45). To confirm that this observation was neither strain-specific nor an epiphenomenon unrelated to the SR, we generated *relA* KO strains in the pathogenic, hyperhemolytic serotype V strain 10/84 (46), and we also generated A909 and 10/84 KO strains lacking *codY* (Δ*codY* strains), which encodes a global transcription factor whose activity is regulated by the balance of GTP and (p)ppGpp and which is a crucial component of the SR (39, 40, 47–50). These additional KO strains all had decreased pigmentation relative to that of the WT in new Granada medium, reflecting decreased βHC expression (Fig. 3A).

We performed hemolysis assays that confirmed functionally that A909 Δ*relA* had decreased cytotoxicity compared to those of the WT and Δ*relA*+pDC123:*relA* strains (Fig. 3B). Next, we performed coincubation studies with GBS and the SR inducer serine hydroxamate (SHX). Dose-dependent βHC expression was easily visualized in 10/84 coincubated with SHX, indicating that chemical SR induction leads to increased βHC production in this strain (Fig. S1). While hyperpigmentation was not observed in A909, a hemolysis assay after SHX coincubation demonstrated increased cytotoxicity (Fig. 3C). The same SHX coincubation was performed with an A909 Δ*cylE* KO strain deficient in βHC expression, which did not lead to increased hemolysis, indicating that the increased βHC expression observed under SR conditions is the cause of enhanced toxicity (Fig. 3C). Coincubation of WT A909 with equimolar concentrations of l-serine, which is chemically similar to SHX, did not enhance hemolysis, nor did SHX in the absence of GBS (Fig. 3C).

To ensure that the SR toxicity effect was not specific to erythrocytes, we studied cytotoxicity against HeLa cells by measuring lactate dehydrogenase (LDH) release after coincubation with A909 Δ*relA* and Δ*codY* strains and observed significantly decreased toxicity from both KO strains (Fig. S2).

**FIG 4:**
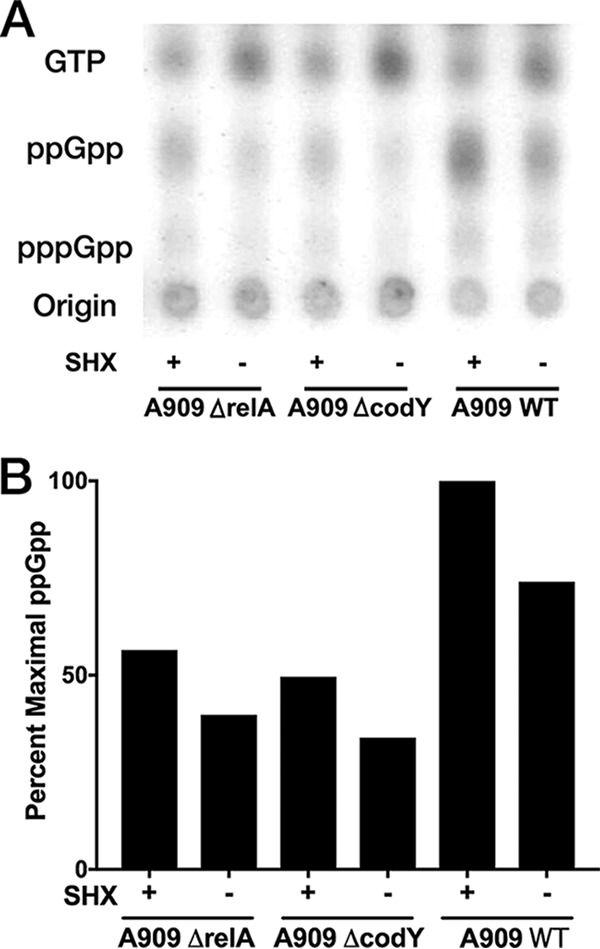
(A) Thin-layer chromatography ppGpp detection from GBS. (B) Densitometry of the ppGpp spots from the autoradiograph presented was performed with ImageJ software. The percentage of maximal ppGpp is relative to the result for the A909 WT SHX-positive condition.

We performed thin-layer chromatography with A909 Δ*relA*, Δ*codY*, and WT strains grown under SR and non-SR conditions to confirm that GBS produces ppGpp, that its levels increase in response to SHX, and that our Δ*relA* strain produces less ppGpp than the WT. We observed decreased but detectable ppGpp levels in both KO strains grown under SR conditions relative to the level in the WT (Fig. 4). This suggests that in GBS, as in other Firmicutes, accessory (p)ppGpp synthases are active, preventing the complete absence of ppGpp in A909 Δ*relA* (51). It also suggests possible direct reciprocal interactions between CodY and RelA or one of the accessory synthases, which would explain the decreased ppGpp levels in A909 Δ*codY*.

### RNA-seq reveals decreased arginine deiminase pathway activity after SR activation.

To explore how SR activation leads to increased βHC expression, we performed whole-genome RNA-seq on RNA isolated from GBS grown in the presence of SHX or in TSB. We used A909 and the hyperhemolytic strain 10/84. This approach also allowed us to compare baseline gene expression differences between the two strains under non-SR conditions.

Summary data for the RNA-seq run is presented in Data Set S2. Overall, there was excellent coverage of the sequencing reads from both strains under SR and control growth conditions, with mapping to 100% of coding sequences in all replicates and reads per kilobase per million mapped reads (RPKM) scores between 667 and 755.

**FIG 5:**
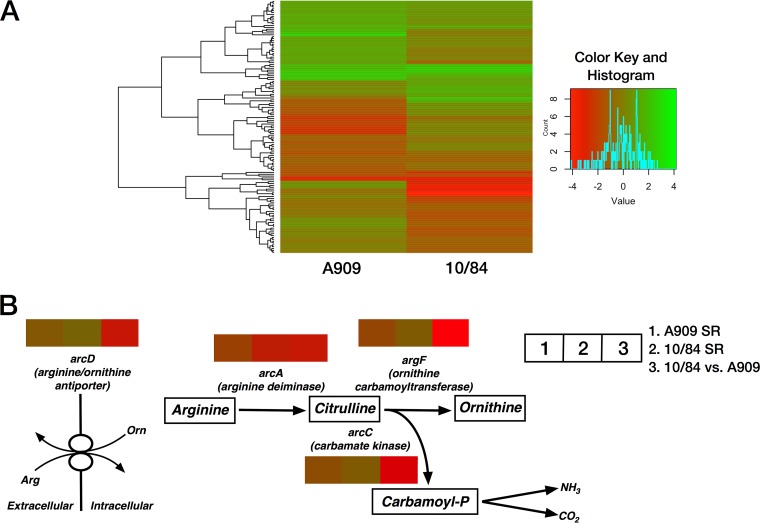
RNA-seq reveals conserved downregulation of the arginine deiminase pathway during SR activation and in comparison between 10/84 and A909 at baseline. (A) Heat map with hierarchical clustering illustrating gene expression changes detected by RNA-seq in A909 and 10/84 during SR activation relative to control growth. All genes that showed >2-fold expression changes (up- or downregulation) in either strain are included. (B) Illustration of the arginine deiminase pathway, with SR versus non-SR and 10/84 versus A909 baseline expression in TSB indicated by the heat-mapped rectangles above each gene in the pathway. The complete list of genes with >2-fold expression changes as a result of SR activation is in Data Set S5. Normalized read counts for cross-strain comparison of A909 and 10/84 under SR and non-SR growth conditions are in Data Set S6.

A909 and 10/84 showed significant between- and within-strain variation in overall gene expression under control and SR conditions, with approximately balanced up- and downregulation of genes induced by SHX (Fig. 5A). We performed gene set enrichment analysis of genes whose transcription was significantly up- or downregulated by SR growth (52). This demonstrated that arginine deiminase pathway genes were significantly overrepresented among the set of genes downregulated by SR in both strains. Furthermore, when we compared whole-genome expression between 10/84 and A909 grown under non-SR conditions, we identified arginine deiminase pathway genes as significantly more highly expressed in A909 than in 10/84 (Table 2).

**TABLE 2:** Gene set enrichment analysis for KEGG classes significantly differently expressed at baseline or following stringent response activation in 10/84 and A909

Strain(s) and condition, KEGG class downregulated	No. of hits	Class size	*P* value	Adjusted *P* value	Description
10/84 vs A909 at baseline					
330	6	12	6.10E−06	0.00011	Arginine and proline metabolism: *arcA*, *argF*, *arc*, *argG*, SAK_2064 (putative duplicated *arcC*), SAK_2065 (putative duplicated *argF*)
500	6	35	5.20E−03	0.03334	Starch and sucrose metabolism
790	3	8	5.60E−03	0.03334	Folate biosynthesis
920	2	3	7.40E−03	0.03334	Sulfur metabolism
190	2	4	1.40E−02	0.04309	Oxidative phosphorylation
A909 under SR^*a*^					
330	2	12	0.021	0.037	Arginine and proline metabolism: *arcA*, *argF*
564	2	6	0.005	0.024	Glycerophospholipid metabolism
640	2	10	0.015	0.034	Propanoate metabolism
650	2	7	0.007	0.024	Butanoate metabolism
1084 under SR					
220	2	9	0.0017	0.0017	Arginine biosynthesis: *arcA*, *glnA*
240	6	42	<1E−12	<1E−12	Pyrimidine metabolism
250	4	13	<1E−12	<1E−12	Alanine, aspartate, and glutamate metabolism

a SR, stringent response.

Combining the results of these analyses suggested that SR may trigger downregulation of genes in the arginine deiminase pathway, which converts arginine to ornithine and carbamoyl-phosphate (Fig. 5B). The fact that we observed the same pattern of relative arginine deiminase pathway downregulation in 10/84, which overproduces βHC compared to its production by A909, suggested that changes in arginine deiminase pathway activity, as well as the resultant changes in intracellular arginine levels, might represent a common mechanism of GBS βHC regulation.

### Arginine availability modulates GBS βHC expression.

**FIG 6:**
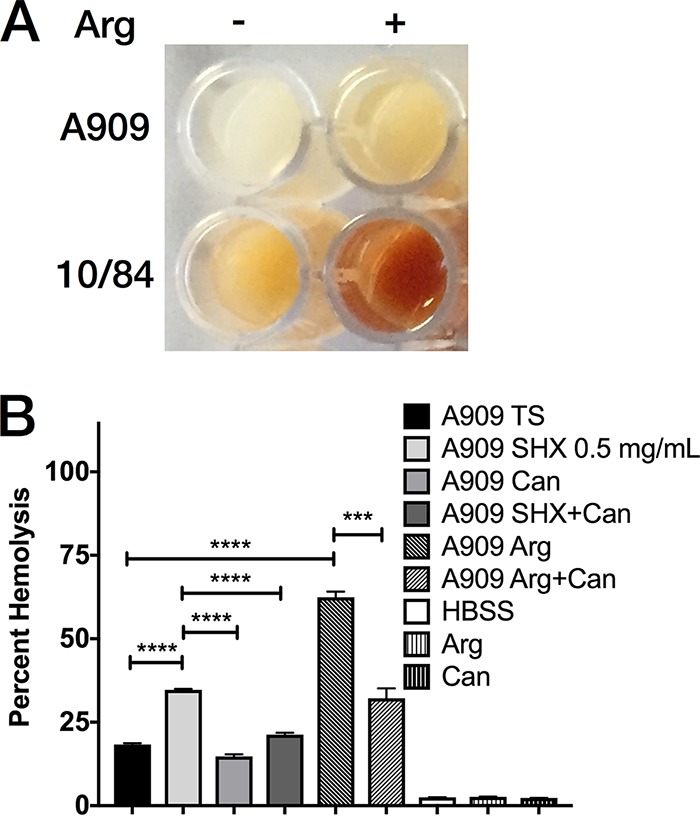
Arginine availability modulates βHC expression and the hemolytic response induced by the SR. (A) A909 and 10/84 WT were grown overnight in TSB with 50 mM arginine or vehicle control. The cultures were normalized for OD_600_ and volume, pelleted, resuspended in 100 μl PBS, and photographed in a 96-well plate. (B) A909 grown overnight with supplemental arginine (10 mM) demonstrates increased hemolytic activity, which is reversed by canavanine (1 mM). Canavanine also reverses the increased cytotoxicity induced by SR induction with SHX. The hemolysis experiment was performed in triplicate and repeated twice. The percentage of hemolysis is relative to the result for a 1% Triton X-100 positive-control solution. Histogram bars show mean values, with error bars illustrating standard errors of the means. ***, *P* < 0.005, and ****, *P* < 0.0001, *t* test with Bonferroni correction for multiple comparisons.

We performed coincubation studies to test the hypothesis that arginine availability is a regulator of GBS βHC expression. The effect of supplemental arginine on GBS βHC was grossly visible, with increased pigmentation under the coincubation condition relative to that in the control; the effect was seen for strains A909 and 10/84 (Fig. 6A).

To test for a functional effect of arginine availability, we coincubated A909 with arginine and the competitive arginine inhibitor canavanine and then performed hemolysis assays with the resultant GBS samples (53, 54). We observed increased hemolysis by GBS grown in the presence of arginine and suppression when canavanine was included in the coincubation. Neither arginine nor canavanine alone had any hemolytic effect (Fig. 6B).

To investigate whether the increased hemolysis observed after induction of the SR depends on arginine availability, we repeated the coincubation with SHX, this time adding an SHX-plus-canavanine condition. We found that 1 mg/ml SHX and 1 mM canavanine was lethal to GBS (data not shown), and so for this experiment, we lowered the SHX concentration to 0.5 mg/ml, which still triggered increased hemolysis while allowing GBS to grow to normal density when canavanine was added. Functionally limiting arginine availability with canavanine reduced the enhanced hemolysis observed with SHX coincubation, suggesting that the mechanism by which the SR leads to increased βHC depends on arginine availability (Fig. 6B).

## DISCUSSION

GBS is a common human commensal organism, with persistent rectovaginal colonization occurring in approximately 25% of asymptomatic adults (55). In order to cause neonatal sepsis, GBS must traverse distinct microenvironments, potentially including cervical mucus, amniotic fluid, the respiratory mucosa, and blood (56). Pathogenesis may also require GBS to survive transcellular host cell passage (7). We have shown that the SR may contribute to GBS pathogenicity in two ways: by enhancing resistance to killing in the bloodstream and by increasing the expression of the pigmented cytotoxin βHC. To our knowledge, this is the first published investigation of the GBS SR.

Our Tn-seq analysis of GBS grown in blood also revealed an important role for the GBS polysaccharide capsule in promoting survival. In addition to serving as a physical barrier against immune factors, the capsule anchors several known surface-associated immune inactivation proteins, such as C5a peptidase and the IgA-binding β antigen, which also emerged from our screen as CE for blood survival (31, 32, 57, 58). Given this background, identification of the *cps* locus as CE for blood survival is not surprising, but supports the validity of our Tn-seq system. Two other genes identified as CE—the R3H domain-containing protein encoded by SAK_0483 and the carbohydrate transporter subunit encoded by SAK_1895—are not characterized and warrant future study.

The SR can be activated by a variety of environmental stresses, many of which are likely to be encountered in the blood. Nutrient deprivation, exposure to antimicrobial peptides or antibiotics, and phagosome exposure can all activate the SR in Firmicutes related to GBS (23, 44, 59). RelA is likely not the sole enzyme involved in (p)ppGpp homeostasis in GBS. Several accessory RelA-related proteins have been described in closely related Streptococcus species (60, 61), and GBS has homologous genes (data not shown). While we have not performed a comprehensive assessment of the roles of those accessory enzymes for this report, further investigation may be worthwhile in future studies.

Widespread transcriptional changes occur in bacteria when the SR is activated, and the changes that promote GBS survival in blood are likely multifactorial and not limited to the arginine deiminase pathway genes that contribute to increased βHC expression. However, the two effects of SR activation that we report—prolonged survival in blood and increased cytotoxin expression—can both be viewed as adaptive responses to a fundamentally inhospitable environment. Based on our findings, the SR allows GBS to survive longer in blood, while also upregulating a cytotoxin that has been shown to promote invasion across anatomical barriers, potentially allowing the bacteria to escape the bloodstream into a less immunologically active space (5, 14, 62). We did not pursue a firm explanation for why complementation of the *relA* KO strain only provided partial rescue of the WT phenotype, but existing studies suggest that the wild-type stringent response is controlled by a finely tuned and interdependent network of (p)ppGpp synthases and hydrolases, as well as second messengers like CodY (40). We speculate that if the transcription of the *relA* gene off the complementation vector is either slightly less or slightly more than its transcription off the chromosome, dysregulation of the stringent response, with reduced bacterial fitness in challenging microenvironments like blood, may result.

Alterations to amino acid metabolism are consistently among the reported SR-mediated effects (39, 63). It is therefore not surprising that conserved changes in the expression of arginine deiminase pathway genes were observed from transcriptomic analysis of GBS strains A909 and 10/84 grown under SR and control conditions. Alterations in arginine deiminase pathway expression have also been reported in GBS and Streptococcus pyogenes in response to human blood or serum, with available data suggesting that exposure to blood triggers dynamic arginine deiminase pathway expression changes and that, in the case of S. pyogenes, some of those changes are mediated by the SR (35, 47, 64, 65). βHC expression has also been shown to promote GBS survival in human blood and in a murine sepsis model. The likely mechanism is through resistance to phagocytic killing (4).

**FIG 7:**
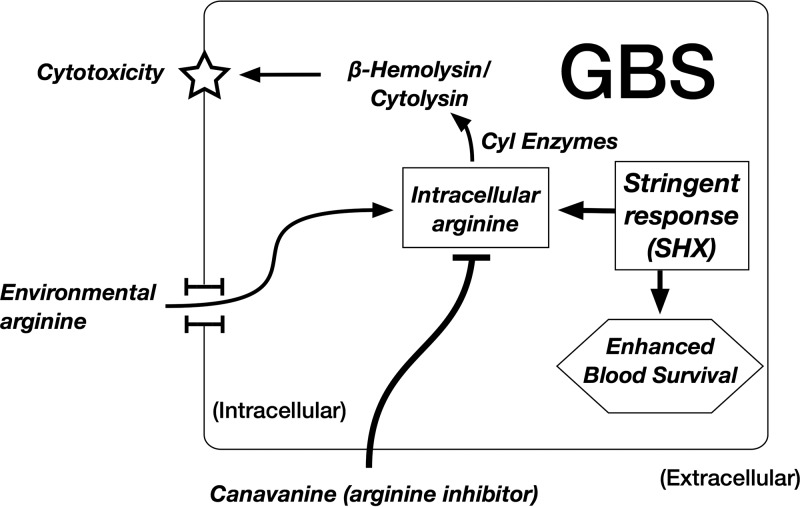
Proposed model of the interaction between SR activation, arginine availability, βHC expression, and cytotoxicity. Based on our data, we believe that GBS arginine availability, which can be functionally limited by canavanine, is a mediator of βHC expression and cytotoxicity. This same arginine-mediated mechanism is activated by the SR, which decreases arginine deiminase activity and also enhances GBS survival in whole blood.

The change in βHC expression by GBS during SR growth was surprising, however. While others have identified a role of the arginine deiminase pathway in controlling the virulence of GBS and related species (35, 66, 67), we are the first to connect SR activation, arginine deiminase pathway expression changes, and βHC regulation. Our proposed model of how intracellular arginine availability regulates GBS virulence through βHC expression is presented in Fig. 7. At this point, we do not know whether arginine feeds directly into βHC biosynthesis or whether it acts in a moonlighting capacity as a signaling factor (68). Given that the proposed βHC biosynthetic pathway does not include arginine, we suspect the latter mechanism (69). We note that the expression of multiple *cyl* genes was upregulated in our SR-versus-control transcriptomic analysis, as well as in the comparison between baseline 10/84 and A909 gene expression levels. The *cyl* operon is known to be regulated by the CovR/S two-component system (70), but its regulation could also be affected by arginine. This is another potential topic for future study.

This work suggests multiple topics for translational investigations of candidate drugs or vaccines. Small molecule inhibitors of the SR have been developed as antimicrobials and have shown some efficacy in preclinical trials, although there are concerns about off-target side effects (71–73). There have been no studies of SR inhibitors for the prevention or treatment of GBS infection. Although canavanine effectively reduced GBS βHC expression in this study, its toxicity profile limits its potential as an antimicrobial (74). However, other, nontoxic arginine antagonists might prove efficacious against GBS infection. Finally, the set of genes that did not meet the strict threshold for being CE in our study but which had decreased transposon insertions suggestive of near essentiality (Data Set S1 in the supplemental material) represents a source of hypotheses about potential drug targets.

In summary, we have presented evidence of a previously unknown virulence pathway in GBS, by which SR activation triggers changes in intracellular arginine homeostasis. Increased arginine availability, in turn, leads to upregulation of the cytotoxin βHC. We believe this work has implications for GBS pathogenesis and, potentially, drug development.

## MATERIALS AND METHODS

### Bacterial strains and growth conditions.

GBS strains A909 (serotype Ia, sequence type 7) and 10/84 (serotype V, sequence type 26) and their derivatives were grown at 37°C under stationary conditions in TSB (product number DF0370-17-3; Fisher Scientific) or new Granada medium (45), supplemented with 5 μg/ml Erm and 5 μg/ml chloramphenicol (Cm) as needed for selection. For the ppGpp detection experiments, GBS was grown on Luria broth (LB) plates to avoid exposing the bacteria to the higher levels of unlabeled phosphorus present in TSB. Escherichia coli was grown at 37°C with shaking in LB medium supplemented with 200 μg/ml Erm and 20 μg/ml Cm as needed for selection.

### Human whole blood collection.

Volunteers for phlebotomy were enrolled after providing informed consent under an active, approved New York University IRB protocol (i15-01305). Antecubital fossa phlebotomy was performed on three healthy adults. Filter-sterilized aqueous heparin sodium salt was added to a final concentration of 2.7 mU/ml. The heparinized blood was used immediately for GBS coincubation.

### Mutant library coincubation with human whole blood.

The Tn-seq-compatible transposon mutant libraries A2, A5, and A7, which are in an A909 background and whose generation has been described previously, were thawed from 2-ml amounts of glycerol stocks stored at −80°C and combined (19). The pooled library was washed three times in 10 ml phosphate-buffered saline (PBS) and then suspended in 3 ml PBS. The 3-ml resuspended library was used to seed 100 ml of TSB Erm, which was grown overnight at 37°C.

The next morning, 20 ml of the overnight library outgrowth culture was used to seed 500 ml of fresh TSB Erm, which was grown to an optical density at 600 nm (OD_600_) of 0.8 (mid-log phase). Sixty milliliters was removed, pelleted by centrifugation, washed once with 20 ml PBS, and then pelleted again and resuspended in 2.5 ml PBS. Serial dilutions of this preparation were plated to determine the initial inoculum, and then 400 μl was used to seed each of five freshly drawn blood samples to 1.5 (10^7^) CFU/ml. A separate 100-ml control outgrowth culture of TSB Erm was seeded with 400 μl and kept at 37°C.

The seeded blood samples were incubated on a rotary mixer at 37°C for 6 h. After coincubation, each blood sample and the control culture were serially diluted and plated to determine colony counts. Five hundred-microliter amounts of each blood sample and the control culture were used to seed outgrowth cultures in 500 ml prewarmed TSB Erm, which were grown under stationary conditions at 37°C overnight.

### Tn-seq DNA preparation and sequencing.

Thirty milliliters from each coincubation outgrowth culture was pelleted and resuspended in 150 μl PBS. Genomic DNA was purified using the MoBio PowerSoil kit according to the manufacturer's instructions. The DNA yields were 15 to 46 ng/μl as measured by spectrophotometry.

Purified DNA was digested with MmeI and ligated to barcoded adapters as described previously (21). Data Set S3 in the supplemental material lists the barcodes used for each of the five experimental samples and the library outgrowth control. Selective PCR amplification of transposon-genome junctions was performed using primers Illumina PCR Tn F and Illumina PCR Adapt R. PCR was limited to 20 to 26 cycles in order to remain in the linear phase of template amplification. Following PCR and agarose gel electrophoresis, the expected 189-bp band was excised and gel extracted using the Qiagen QIAquick kit. Purified samples were assessed on an Agilent Bioanalyzer before sequencing. Amplicon samples were multiplexed and sequenced on a 150-nt paired-end run of the Illumina HiSeq 4000 platform, with a target number of reads per library of ∼50 million. Demultiplexing and read binning were performed using the open source tool FastqMultx (75).

### Determination of conditionally essential genes.

Demultiplexed Illumina reads were trimmed of flanking adapter and transposon sequences using the open source tool Cutadapt (76). GBS-specific sequences that were <12 nt or >25 nt were discarded. The remaining sequences were aligned to the A909 genome (GenBank accession number NC_007432) using BowTie2 (77).

The resultant BAM files were uploaded to ESSENTIALS and analyzed with the following parameters: Loess genomic position bias off, read count normalization with TMM (trimmed mean of M values), data dispersion estimation with qCML (quantile-adjusted conditional maximum likelihood), and tagwise modeling of variance with amount of smoothing set to 5. The expected versus experimental insertion density fold change cutoff generated by ESSENTIALS was used to separate CE from nonessential genes. The TA site insertion tallies for the data in Fig. 1 were generated using ARTIST (78).

### Generation of GBS mutants.

The vector insertion mutant GBS Δ*relA* was generated using the temperature-sensitive shuttle vector pHY304 as previously described (79), with the following details. pHY304 was linearized with SpeI and gel extracted. A 500-bp internal fragment of the A909 *relA* gene with terminal overhangs complementary to the pHY304 free ends, generated by SpeI digestion, was amplified using PCR primers relA_intF and relA_intR. The fragment was gel extracted and combined with the purified digest of pHY304 in a Gibson assembly reaction mixture to make pHY304:*relA*_frag, which was subsequently used to transform chemically competent E. coli DH5α with Erm selection at 28°C. Transformants were confirmed by PCR using pHY304 primers that flank the intended insertion site (pHY304_mcsF and PHY304_mcsR).

Miniprepped plasmid samples were then used to transform electrocompetent A909 with TSB Erm selection at 28°C as described previously (19). We used PCR with pHY304_mcsF and PHY304_mcsR to confirm that the resultant colonies carried the intended plasmid. Single-cross vector insertion mutants were generated by transitioning liquid cultures of transformed A909 from 28°C to 37°C at early to mid-log growth while under Erm selection. Correct insertion of the plasmid in the *relA* gene was confirmed with PCR using relA_outsideF and pHY304_mcsF, followed by Sanger sequencing with pHY304_mcsF to confirm that the expected pHY304-A909 genome junction had the predicted sequence (data not shown).

A909 Δ*codY* and Δ*cpsE* were generated using analogous procedures with the corresponding PCR primers listed in Data Set S4. For A909 Δ*cpsE*, successful disruption of the *cpsE* gene was functionally confirmed based on a negative GBS serotype Ia latex agglutination test rather than PCR (data not shown).

To generate the complemented Δ*relA*+pDC123:*relA* strain, the shuttle vector pDC123 was linearized with BamHI. The A909 *relA* gene and its promoter were amplified with the primers relA_compF and relA_compR, which have appropriate overhanging sequences complementary to pDC123. The plasmid and insert were ligated with Gibson assembly and transformed into E. coli DH5α with Cm selection. After PCR confirmation of successful transformation, the plasmid was miniprepped and used to transform electrocompetent A909 Δ*relA* with selection on TSB agar Erm plus Cm. Successful transformation of the clone used in experiments was confirmed by PCR.

A909 Δ*cylE* was a generous gift from Victor Nizet. This strain features allelic replacement of the *cylE* gene with the Cm resistance cassette *cat*, generated using established methods (79). It exhibits weak Cm resistance, so it was grown on TSB agar without selection.

### Thin-layer chromatography for ppGpp detection.

The method described by Cashel for (p)ppGpp detection from E. coli was adapted as follows (80). A909 WT, Δ*relA*, and Δ*codY* were grown overnight on LB plates with appropriate selection. Individual colonies were scraped from the agar and resuspended in 5 ml MOPS (morpholinepropanesulfonic acid) minimal medium without supplemental phosphorus or serine to achieve an OD_600_ of 0.9. Each bacterial suspension was then divided into two 65-μl aliquots, to which 10 μl ^32^P was added for a final concentration of >100 mCi/ml. For the SR activation conditions, SHX in MOPS was added to a final concentration of 1 mg/ml; control samples were spiked with an equal volume of MOPS without SHX. After 30 min, one volume of 13 M formic acid was added to the samples, which were then subjected to three sequential freeze-thaw cycles. The bacterial debris was pelleted by centrifugation, and the supernatants spotted to polyethyleneimine cellulose-coated thin-layer chromatography plates, where they were allowed to dry. The plates were run in covered beakers with 1.5 M KH_2_PO_4_. Once this buffer was near the top of the plate, the plate was dried and exposed to autoradiography film overnight, which was then developed and photographed.

### Blood coincubation with KO strains.

Fifty-milliliter cultures of GBS were grown in TSB with appropriate selection overnight. The cultures were normalized to an OD_600_ of 1.0, and then 30-ml aliquots were pelleted and washed once with 10 ml PBS. After repeat centrifugation of the washed bacteria, the pellet was resuspended in 2.5 ml of PBS, and serial dilutions of this suspension were plated to determine the input inoculum.

Four hundred microliters of the bacterial suspension was injected into 4 ml of freshly drawn, heparinized human whole blood, which was maintained on a rotary mixer for 4 h, at which point serial dilutions were plated on TS agar for colony counts. Fractional survival for each sample was calculated as the output CFU concentration divided by the starting CFU concentration.

### Stringent response RNA-seq.

Amounts of 150 μl of overnight cultures of A909 and 10/84 in TSB were used to seed 45 ml of prewarmed, filter-sterilized TSB with or without 1 mg/ml SHX. Each condition was tested in triplicate. The cultures were grown under stationary conditions until they reached an OD_600_ of 0.6. Whole RNA was then purified from 13-ml samples using the Ambion RiboPure bacterial kit according to the manufacturer's instructions. RNA samples were treated with DNase twice, for 1-h intervals, with enzyme inactivation between treatments. Samples were analyzed on an Agilent bioanalyzer, which demonstrated a mean RNA concentration of 162 ng/μl and a mean RNA integrity number (RIN) of 9.4. rRNA was removed with Illumina Ribo-Zero treatment according to the manufacturer's instructions. Enriched mRNA was fragmented and used for synthesis of strand-specific cDNA using the NEBNext Ultra directional RNA library preparation kit (NEB catalog number E7420L). The DNA was purified between enzymatic reactions, and size selection of the library performed with AMPure SpriSelect Beads (Beckman Coulter Genomics). The titers and sizes of the libraries were assessed on the LabChip GX (PerkinElmer) and with the library quantification kit for Illumina (Kapa Biosciences). Libraries were sequenced on the Illumina HiSeq 2500 platform using 125-nt paired-end reads, with a target of 40 million reads per library. Following demultiplexing, sequences were aligned to the reference A909 (GenBank accession number NC_007432) and 10/84 (GenBank accession number NZ_CP006910.1) genomes using Bowtie version 0.12.9. Genes with a significant treatment effect (up- or downregulation in SHX) were determined with DESeq version 1.10.1 (with the following cutoffs: *P* value, ≤0.05; read count percentile, ≥0.25; and fold change, ≥2).

For A909 versus 10/84 transcriptome comparisons, RNA-seq results from the two strains grown under SR and non-SR conditions were normalized for read numbers. Orthologous genes shared by the two strains were identified using the CloVR-Comparative pipeline (81, 82) and Sybil (83). Normalized read counts for orthologs were compared directly using in-house scripts.

### Hemolysis assays.

Fifty-milliliter cultures of GBS were grown in TSB or new Granada medium with appropriate selection and the additives indicated in Fig. 3 and 6, after confirming that none of the additives changed the broth pH from ∼7.0. Additive concentrations were 1 mg/ml (0.83 μM) or 0.5 mg/ml (0.42 μM) SHX, 0.83 μM l-serine, 10 mM arginine, and 1 mM canavanine. The cultures were normalized to an OD_600_ of 1.0, and then 30-ml aliquots were pelleted and washed once with 10 ml PBS. After repeat centrifugation of the washed bacteria, the pellet was resuspended in 1 ml of PBS, and serial dilutions of this suspension were plated to determine the CFU concentration (mean = 2 × 10^8^ CFU/ml). This sample was diluted 1:50 and combined 1:1 with a preparation of 1% washed, packed human erythrocytes in Hanks' buffered saline solution (HBSS). Coincubation was for 90 min. Hemolysis rates were determined by spectrophotometric measurement of free hemoglobin using established methods (84). For additive-only negative controls, the same protocol was followed, replacing GBS with sterile additive dissolved in PBS to the concentrations indicated in Fig. 3 and 6. Hemolysis (OD) is reported as the percentage relative to the result for treatment with a 1% solution of Triton X-100 in HBSS.

### LDH assay.

Human cervical epithelial cell cultures (HeLa, ATCC CCL2) were grown to confluence in 24-well plates at 37°C and 5% CO_2_ in Eagle's minimum essential medium with supplemental fetal bovine serum (FBS), sodium pyruvate, and ciprofloxacin following standard protocols. GBS strains were grown in TSB with appropriate Erm selection for KO strains. At an OD_600_ of 0.6 (mid-log phase), bacteria were pelleted, resuspended in RPMI medium, and adjusted to achieve a multiplicity of infection of 10. Serial dilutions were plated on TS agar to confirm the correct CFU concentration (data not shown). Prior to coincubation, the HeLa cells were washed three times with RPMI medium without added supplements (15 min per wash). Amounts of 500 μl of GBS samples were added to the experimental wells. RPMI medium alone served as the negative control, while 1% Triton X-100 in RPMI medium was the positive control. The cell plate was spun at 200 relative centrifugal force (RCF) for 2 min and then returned to 37°C and 5% CO_2_ for 4 h. Following coincubation, 300 μl supernatant from each well was analyzed for LDH concentration using the Roche cytotoxicity detection kit (catalog number 04744926001), following the manufacturer's instructions.

### Statistics and data visualization.

*t* tests with Bonferroni corrections for multiple comparisons were performed using GraphPad Prism 7.0. The Tn-seq Circos plot (Fig. 1) was generated using Circos 0.67-7 (26). Heat map data for Fig. 5 were generated using HeatmapGenerator 5.0 (85). Gene set enrichment analysis for the data in Table 2 was performed with Genome2D (52).

### Availability of data.

RNA-seq reads are available under GEO accession number GSE98398. Tn-seq reads are available under BioProject accession number PRJNA416503.

## Supplementary Material

Supplemental material
