# Supplementary material for: The Streptococcus agalactiae Stringent Response Enhances Virulence and Persistence in Human Blood
Source: Infect Immun. 2017 Dec 19;86(1):e00612-17. doi: 10.1128/IAI.00612-17 (PMC5736797; doi:10.1128/IAI.00612-17)
Supplement: Supplemental material [file supp_86_1_e00612-17__index.html]

Supplemental material 

# The Streptococcus agalactiae Stringent Response Enhances Virulence and Persistence in Human Blood

## Supplemental material

- Supplemental file 1 -

  Data Set S1. Essentials output from analysis of whole-blood Tn-seq.

  XLSX, 227K
- Supplemental file 2 -

  Data Set S2. Summary data for the RNA-seq runs, including sequencing details for the nine samples included in the RNA-seq analysis (three replicates for A909 and 10/84 TS and SHX growth).

  XLSX, 55K
- Supplemental file 3 -

  Data Set S5. RNA-seq data for A909 and 10/84 showing all genes with >2-fold up- or downregulation after treatment with SHX.

  XLSX, 83K
- Supplemental file 4 -

  Data Set S6. RNA-seq data for A909 and 10/84 showing homologous genes with normalized read counts for growth in TS and SHX.

  XLSX, 353K
- Supplemental file 5 -

  Fig. S1. Changes in βHC pigmentation in 10/84 and A909 after overnight growth in TS media with supplemental SHX. Fig. S2. Cytotoxicity of A909 ΔrelA and ΔcodY as determined by LDH release from HeLa cells following coincubation. Caption for Data Set S1. Caption for Data Set S2. Data Set S3. Expected Tn-seq amplicon sequence with key features annotated, and barcodes used for samples sequenced in the present study. Data Set S4. Plasmids and PCR primers used in the present study. Caption for Data Set S5. Caption for Data Set S6.

  PDF, 4.0M
