# Supplementary material for: The Streptococcus agalactiae Stringent Response Enhances Virulence and Persistence in Human Blood
Source: Infect Immun. 2017 Dec 19;86(1):e00612-17. doi: 10.1128/IAI.00612-17 (PMC5736797; doi:10.1128/IAI.00612-17)
Supplement: Supplemental material [file IAI.00612-17_zii999092272s5.pdf]

Supplemental figures and datasets

Supplemental Figure S1 Changes in βHC pigmentation in 10/84 and A909 after overnight growth in TS media with supplemental SHX. Cultures were normalized for OD_{600} and volume, pelleted, resuspended in 100 µL PBS, and photographed in a 96-well plate. L-serine at an equimolar concentration to SHX 1 mg/mL was included as a negative control.

Supplemental Figure S2 Cytotoxicity of A909 ΔrelA and ΔcodY as determined by LDH release from HeLa cells following coincubation. Percent cytotoxicity is reported relative to the 1% triton X-100 positive control. Each condition was tested in triplicate. Bars indicate mean values with error bars showing standard error of the mean. * $p < 0.05$, T-test with Bonferroni correction for multiple comparisons.

Supplemental Dataset S1 ESSENTIALS output from analysis of whole blood Tn-seq. Data presented for each gene locus includes LFC value for experimental samples (column C); the non-adjusted and adjusted p values (columns D-E) from an exact test based on the negative binomial distribution model in EdgeR incorporated in the ESSENTIALS package. Columns F-J provide details on gene location, orientation, and annotation (if any). The table is sorted by LFC (from smallest to largest). NA=not available; note that essential genes (i.e. necessary for GBS growth in broth) will produce NA when conditional essentiality is determined because there are too few transposon insertions in the control condition to perform a statistical comparison.

Supplemental Dataset S2 Summary data for the RNA-seq runs, including sequencing details for the nine samples included in the RNA-seq analysis (three replicates for A909 and 10/84 TS and SHX growth). Details listed include: total paired-end reads (column B); total mapped reads (column C); percent mapped reads (column D); percent of reads properly paired (column E); percent of reads that mapped to intergenic regions (column F); total genes with complete coverage (column G); and average reads per kilobase per million mapped reads (column H).

Supplemental Dataset S3 Expected Tn-seq amplicon sequence with key features annotated, and barcodes used for samples sequenced in the present study.

Supplemental Dataset S4 Plasmids and PCR primers used in the present study.

Supplemental Dataset S5 RNA-seq data for A909 and 10/84 showing all genes with >2-fold up- or downregulation after treatment with SHX. P values were determined by DESeq v1.10.1 (with the following cutoffs: p-value ≤0.05, read count percentile ≥0.25 and fold change ≥2).

Supplemental Dataset S6 RNA-seq data for A909 and 10/84 showing homologous genes with normalized read counts for growth in TS and SHX. Tabs 3 and 4 show unique genes in each of the two genomes, with associated normalized read counts.

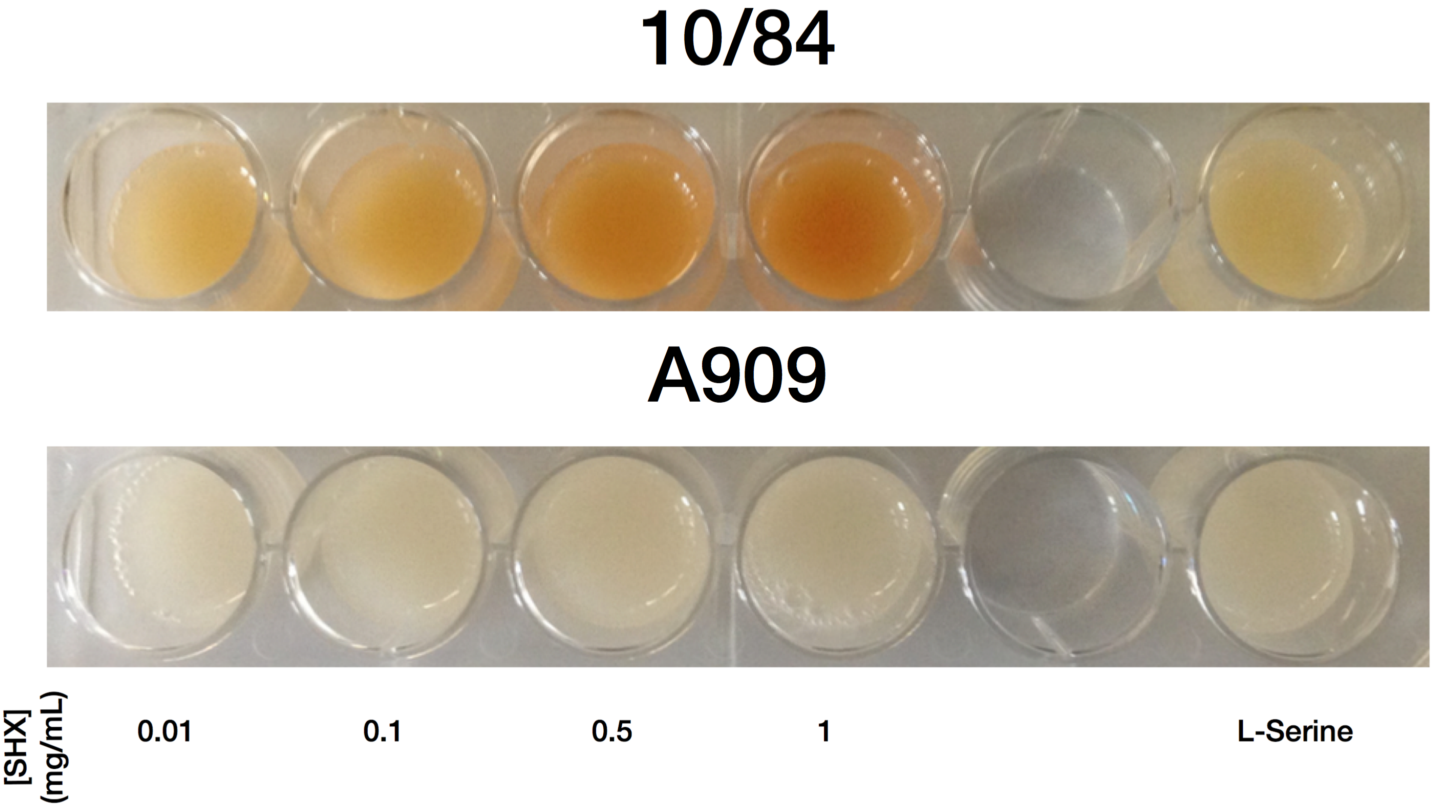

Fig. S1

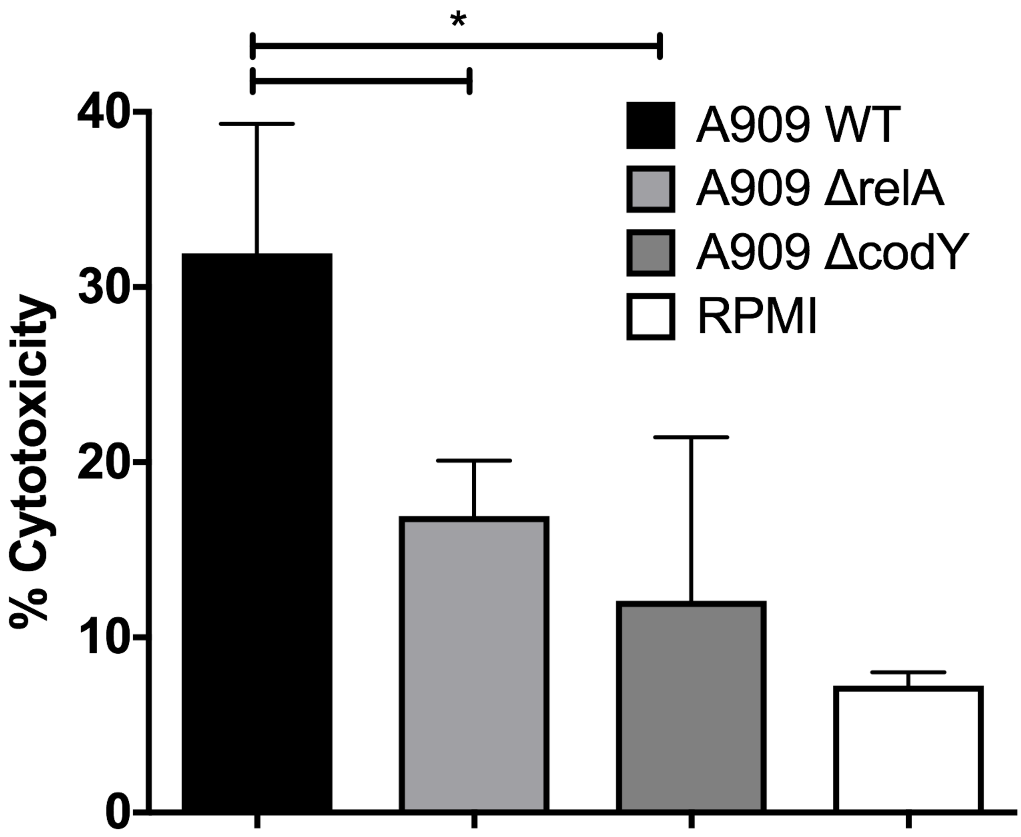

Fig. S2

Supplemental Dataset S3.

Expected fragment characteristics:

189 bp

AATGATACGGCGACCACCGAGATCACACTCTTTCCCTACACGACGCTCTTCCGATCT*XX*
*XXXXXX***NNNNNNNNNNNNNNTA**ACAGGTTGGATGATAAGTCCCCGGTCTGACACATAGA
TGGCGTCGCTAGTATTAAATGCAGTAGATCCGAAGATCAGCAGTTCAACCTCGTATGCC
GTCTTCTGCTTG

Single Underline: Illumina-specific sequence
XXX: Barcode
Bold: GBS sequence (from strain A909)
Double Underline: MmeI binding site

Samples and Barcodes:

H1	ATAGAGGC
H2	CCTATCCT
C1	AGATGAAA
C2	AGCAAATA
R2	ATGAAGGC
Control	TATAGCCT

Dataset S4. Plasmids and Primers used in the present study.

Plasmid/Primer	Sequence	Reference
pCAM48	GenBank: KU936423.1	(1)
pHY304	GenBank: KU936424.1	(2)
pDC123	GenBank: AF167172.1	(3)
Illumina PCR Tn F	CAAGCAGAAGACGGCATACGAGGTTGAA CTGCTGATCTTCGG	(1)
Illumina PCR Adapt R	AATGATACGGCGACCACCGAGATCACAC TCTTTCCCTACACGACGCTCTTCC	(1)
relA_intF	AGCCCGGGGGATCCAGCTTTTTCTCCAAC TTGC	Present study
relA_intR	GCGGCCGCTCTAGAACAGATTTATGATTT AATTGCG	Present study
pHY304_mcsF	CAATACGCAAACCGCCTCTC	Present study
PHY304_mcsR	AAAGAGGTCCCTAGCGCCTA	Present study
relA_outsideF	TCTTCTGGAGCAATCCCAACT	Present study
relA_compF	TTACTAGGATCCTGCAGCTGAATTCTAAC TTCTTTTATTTTTAGGTTAAAAAG	Present study
relA_compR	TCTAGATATCGATGCATATGAATTCTAAC CATTCGTACGTTTAAC	Present study
codY_intF	AGCCCGGGGGATCCAGTTAAGTAATTGA ATACCAACAAC	Present study
codY_intR	GCGGCCGCTCTAGAATTACTATGTTAAAT CTGCTAGTCG	Present study
codY_outsideF	ACTCCCAGTCCCATGCTATG	Present study
cpsE_intF	AGCCCGGGGGATCCACTAGTAATACAAG ATATTATTAATGACATTGAAG	Present study
cpsE_intR	GCGGCCGCTCTAGAACTAGTATCACCCTT TAAAACATTATAGAATTG	Present study

Supplemental References

1. **Hooven TA**, **Catomeris AJ**, **Akabas LH**, **Randis TM**, **Maskell DJ**, **Peters SE**, **Ott S**, **Santana-Cruz I**, **Tallon LJ**, **Tettelin H**, **Ratner AJ**. 2016. The essential genome of Streptococcus agalactiae. BMC Genomics **17**:406–418.

2. **Yim HH**, **Rubens CE**. 1998. Site-specific homologous recombination mutagenesis in group B streptococci. Methods in Cell Science **20**:13–20.

3. **Chaffin DO**, **Rubens CE**. 1998. Blue/white screening of recombinant plasmids in Gram-positive bacteria by interruption of alkaline phosphatase gene (phoZ) expression. Gene **219**:91–99.
